# Domestic Correspondence

**Published:** 1889-02

**Authors:** 


### Domestic Correspondence.

To the Editor:

In an answer to your letter of inquiry relative to the organization and aims of, as you term it, the "phenominally developed society," will say that the Odontological Society of Pennsylvania was organized January 18, 1879, and the first meeting held Saturday evening, February 1, 1879. *No charter.*

The aims of the Society are to diffuse the principles of the art and science of dentistry; to develop new modes of practice, and to give encouragement to inventors of useful instruments, or anything in mechanism pertaining to the practice of dentistry, to originators of new modes of manipulation and superior materials for filling. To add to the literature of the profession, by the reading and publication of essays and discussions thereupon, and incidents of practice. To elevate the profession by admitting to membership none but graduates in dentistry or medicine.

The following gentlemen became members at its organization: Drs. Essig, Guilford, Webb, Woodward, Register, Darby, Tees, Huey, Jack, D. Neall, E. H. Neall, Bonwill, Kingsbury, Longnecker and Dixon.

At present we have 87 active members—two of whom are lady practitioners, 2 honorary members and 3 corresponding members; the latter having been active members but are now practising in other states or countries.

Meetings held monthly.

AMBLER TEES,
Phila., Pa., Jan. 15, 1889. *Secretary.*

---

BALTIMORE, NOV. 1st, 1888.*

*Dr. Fred A. Levy,*
Secy. Nat. Association of Dental Examiners.

*Dear Sir:* I desire to acknowledge the receipt, this day, of a printed copy of the proceedings of the Seventh Annual Meeting of the Nat. Assoc. of Dental Examiners, and, in return, to send you enclosed circulars of the correspondence between Dr. Waters, Dr. G. F. S. Wright and myself, and also a letter from Dr. W. C. Wardlaw, of the Georgia State Board of Dental Examiners, who with Dr. S. B. Barfield, represented said board at the Louisville meeting of your association. According to Dr. Wardlaw's letter, the list of dental schools were reviewed and re-reviewed two or three times, and finally disposed of, with no objections to the "University of Maryland Dental Department," which as Dr. Wright also states, was "*passed without objection or comment of any kind.*"

In your report of the proceedings (according to the printed pamphlet received to-day), it appears that in the last session of your association, at which there was a very much reduced attendance from the *seventeen* original representatives, but at which session, the State Board of Georgia *was* represented, if that of South Carolina was not, the list of dental schools was again revised and, according to the number published in your printed proceedings, the University of Maryland Dental Department was omitted.

Is not such a record of your proceedings strangely incorrect as Dr. G. F. S. Wright, a copy of whose letter I send you, was according to your report, one of the committee appointed to prepare a list of Dental Colleges which your association would recommend to the various State Boards?

Does not Dr. N. C. Wardlaw state that although he was not present at the last session, yet he understood and was "told that nothing had been done but the election of officers and routine business."

Yet your report alleges that the list of colleges was again (not-

---

* Correspondence sent by Dr. Levy, and which was crowded out of January issue.

withstanding the many previous reviews) " referred back to the committeee to be revised, and was again presented to the association and accepted."

The conclusion I arrived at, from all the testimony given in the letters of Drs. Wright and Wardlaw, who declare that the University of Maryland Dental Department was not omitted from the list of Dental Colleges whose diplomas were to be recommended to the various State Boards (the former gentleman being a member of the committees appointed to prepare the said list of Dental Colleges), are that you have either confounded the proceedings of the second session of your association with those of the third session, unintentionally omitting the name of our department by reason of such mistake, or that advantage was taken of the absence of several of the representatives who were present at the former session, to injure, without any just cause, our institution ; for if it is true, which I greatly doubt, that such action was taken at your last session, it could not have been done unanimously at the previous sessions, as the letters of both Dr. Wright and Dr. Wardlaw prove. Respectfully, etc.,

FERDINAND J. S. GORGAS,
Dean of the University of Md., Dental Department.

---

ORANGE, N. J., November 5, 1883.

FERDINAND J. S. GORGAS, M.D., D.D.S., Dean, Baltimore, Md.

*Dear Sir:*—Yours on November 1st, enclosing circulars containing correspondence with Drs. Waters, Wright and Wardlaw received, and in reply I would state :

1st. That I have no record of the attendance of Dr. Wardlaw, and it appears from his own statement that he was not present at the session at which was passed the resolution complained of by you.

2d. Dr. Wright is in error relative to the proceedings of the National Association of Dental Examiners, when he states that your college " passed without objection or comment of any kind." In this important particular : that there was opposition to the insertion of the name of your college among those " whose diplomas to the National Association of Dental Examiners recommended the various State Boards to accept instead of an examination," or it would not have been dropped from the revised list presented by the committee ; for particulars of the passage of this resolution I refer you to page 4 of the printed proceedings of the association, from which it appears that "the list of colleges presented and accepted at the last meeting was re-considered. The list was then referred back to the committee to be revised, and was again presented to the association; was amended and accepted," omitting the name of your college.

3d. That this *report* of the committee, appointed for that purpose, consisting of Drs. Wright, Rawls and Levy, was *signed* by each of them, and in that form presented to the association; this *original* report, omitting the name of your college, and signed by Dr. Wright with the others mentioned, I have in my possession, which you can see upon application.

4th. At the time of the passage of this resolution, as appears upon page 4 of the proceedings mentioned, the roll-call showed ten states represented.

5th. The printed report of the proceedings of the association sent you are correct in every particular, the assertion of Dr. Wardlaw (who was not present), and those of Dr. Wright (who concurred in the resolution dropping your college), to the contrary notwithstanding. As you will see the conclusions which you arrive at, from what you choose to term the testimony in your possession, are totally incorrect, inasmuch as the name of your college was intentionally omitted from the list of the Dental Colleges which the National Association recommended the various State Boards, whose diplomas might be accepted instead of an examination.

There has been no confusion upon my part in the matter; upon the contrary I have been exceedingly particular, and know that there has been no mistake whatever.

Any other or further information you may desire I shall be pleased to furnish you. Very truly yours,

Fred. A. Levy, *Secretary*,
of the National Association of Dental Examiners.

---

To the Editor :

*Dear Sir :*—The letter of Dr. Fred. A. Levy, as well as your comments published in the Jan. No. of your Journal, do injustice to both the publisher and editor of the *Am. Journal of Dental Science*, so far as relates to an alleged suppression of Dr. Levy's letter to me. The reason it did not appear in the *Am. Journal of Dental Science* before this time was, that the original copy was sent South to the gentlemen whose veracity it questioned. As a copy of said letter was received from Dr. Levy, it was placed in the printer's hands, and will appear in the forthcoming number. As the *Am. Journal of Dental Science* is a monthly publication, Dr. Levy's letter, under the circumstances, could not appear earlier.

Baltimore, Md., Jan. 11, 1889. F. J. S. GORGAS.

[Our comments on Dr. Levy's letter were made not with the intention of taking sides on the question, but simply to abbreviate the matter sent which it was impossible to get in the January issue.—ED.]

